# Proteomic Characterization of High-Density Lipoprotein Particles from Non-Diabetic Hemodialysis Patients

**DOI:** 10.3390/toxins11110671

**Published:** 2019-11-15

**Authors:** Nans Florens, Catherine Calzada, Frédéric Delolme, Adeline Page, Fitsum Guebre Egziabher, Laurent Juillard, Christophe O. Soulage

**Affiliations:** 1 Univ. Lyon, CarMeN, INSERM U1060, INSA de Lyon, Université Claude Bernard Lyon 1, INRA U1397, F-69621 Villeurbanne, France; catherine.calzada@insa-lyon.fr (C.C.); fitsum.guebre-egziabher@chu-lyon.fr (F.G.E.); laurent.juillard@univ-lyon1.fr (L.J.); 2 Hospices Civils de Lyon, Service de Néphrologie-Hypertension-Hémodialyse, Hôpital E. Herriot, F-69003 Lyon, France; 3 Protein Science Facility, SFR BioSciences CNRS UMS3444, Inserm US8, Université Claude Bernard Lyon 1, ENS de Lyon, F-69007 Lyon, France; frederic.delolme@ibcp.fr (F.D.); adeline.page@ibcp.fr (A.P.)

**Keywords:** HDL cholesterol, lipoproteins, cardiovascular risk, proteomic, mass spectrometry, hemodialysis

## Abstract

Chronic kidney disease is associated with an increased cardiovascular risk, and altered biological properties of high-density lipoproteins (HDL) may play a role in these events. This study aimed to describe the HDL proteome from non-diabetic hemodialysis patients and identify potential pathways affected by the dysregulated expression of HDL proteins. HDL were sampled from nine non-diabetic hemodialysis (HD) and eight control patients. Samples were analyzed using a nano-RSLC coupled with a Q-Orbitrap. Data were processed by database searching using SequestHT against a human Swissprot database and quantified with a label-free quantification approach. Proteins that were in at least five of the eight control and six of the nine HD patients were analyzed. Analysis was based on pairwise ratios and the ANOVA hypothesis test. Among 522 potential proteins, 326 proteins were identified to be in the HDL proteome from HD and control patients, among which 10 were significantly upregulated and nine downregulated in HD patients compared to the control patients (*p* < 0.05). Up and downregulated proteins were involved in lipid metabolism, hemostasis, wound healing, oxidative stress, and apoptosis pathways. This difference in composition could partly explain HDL dysfunction in the chronic kidney disease (CKD) population and participate in the higher cardiovascular risk observed in this population.

## 1. Introduction

Chronic kidney disease (CKD) is associated with an increased cardiovascular risk [1]. The failure of statins to reduce the cardiovascular risk of hemodialysis (HD) patients has recently led researchers to focus on the specific properties of high-density lipoproteins (HDL) among this population [2,3]. HDL are essential lipoproteins that exert several atheroprotective properties, among which are the induction of cholesterol efflux from peripheral macrophages and anti-inflammatory, anti-oxidant, vasoprotective, and anti-aggregant effects [4]. These atheroprotective properties of HDL are found to be deeply impaired in CKD and particularly in HD patients [5]. Indeed, several studies have reported that dysfunctional HDL particles from CKD patients can lead to vasoconstrictive and pro-inflammatory properties [5,6] and a reduction in cholesterol efflux [5,6,7,8].

HDL are also essential for lipid and lipoprotein metabolism and homeostasis [9]. CKD leads to a unique phenotype of dyslipidemia with impaired lipoprotein metabolism, particularly in end-stage renal disease (ESRD) patients. In those patients, there is an accumulation of triglyceride-rich particles, mainly very-low-density lipoproteins (VLDL) and chylomicrons. ESRD patients, however, present with normal or lowered low-density lipoprotein (LDL) levels [10].

HDL protein composition directly determines the particle properties and has already been associated with poor cardiovascular outcomes in non-CKD subjects [11,12]. In ESRD patients, label-free proteomic analysis highlighted that dysregulated levels of certain proteins in HDL particles could lead to impaired biological functions [12,13]. Another study found that increased levels of serum albumin, serum amyloid A, and apolipoprotein C-III in HDL particles were correlated with a decrease in the levels of essential apolipoprotein A1 and A2 [8]. Moreover, an increased level of alpha-1-microglobulin/bikunin precursor (AMBP), β2-microglobulin (B2M), complement factor D (CFD), cystatin-C (CST3), and retinol-binding protein 4 (RBP4) has been reported in HDL particles from ESRD patients [13]. Taken separately, these three studies have demonstrated that the HDL proteome consists of around 100 proteins.

Since HDL proteome composition can directly influence the biological functions of this lipoprotein, we chose to use a high-resolution mass spectrometry analysis and label-free quantification approach to investigate the proteomic composition of HDL isolated from non-diabetic HD patients. Such quantification of HDL protein composition should be useful in identifying new markers of HDL dysfunction and assessing cardiovascular risk among HD patients.

## 2. Results

Eight healthy controls (CTL) were compared to nine non-diabetic HD patients, the main characteristics of whom are presented in Table 1. HD patients exhibited significantly lower total cholesterol, LDL-cholesterol, and HDL cholesterol than the controls (*p* < 0.05). Two-thirds of HD patients were under statin therapy.

### 2.1. Proteome Characteristics

**Figure 1:**
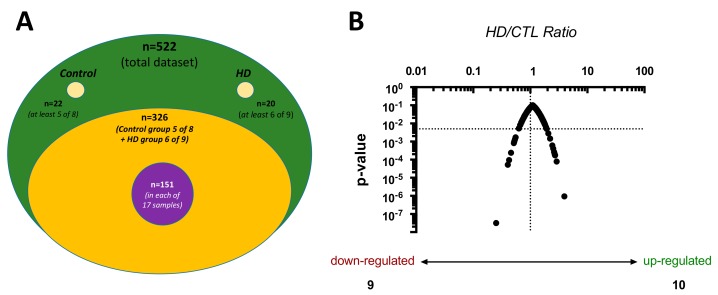
Among 522 proteins, 326 were at least found in 60% of the samples (id. five of the eight controls and six of the nine hemodialysis (HD) patients). Twenty-two and 20 proteins were only found in control and HD samples, respectively. Among the 522 proteins, 151 were found in every sample. (**A**). The hemodialysis/control patient (HD/CTL) protein ratio was calculated as the protein abundance in HD patients divided by protein abundance in control patients. Among 326 proteins, nine were significantly downregulated while 10 were upregulated (see Table 2 and Table 3 for details). *P* < 0.05 was considered as significant (dot line, **B**).

Five hundred and twenty-two (522) proteins were identified from the mass spectrometry analysis of both control and HD patients, among which 482 were found in more than one patient (Table S1). To avoid potential plasma contamination, we decided to only analyze the proteins that were found at least in 60% of the patients (id. five out of the eight controls and six out of the nine HD patients). Three hundred and twenty-six (326) proteins were found in both conditions (Figure S1), among which 151 were found in the 17 samples. Among those 326 proteins, 10 were significantly upregulated while nine were significantly downregulated in HD patients compared to control patients (*p* < 0.05, Figure 1, Table 2 and Table 3). Twenty-two (22) proteins were only found in control samples (Table S2) and 20 in HD samples (Table S3). Regarding the HDL proteome watch database (http://homepages.uc.edu/~davidswm/HDLproteome.html), we recovered 69 of the 95 (73%) proteins defined as appearing in three separate studies from three independent laboratories. We also found 155 of the 228 proteins cited in this database (70%).

**Figure 2:**
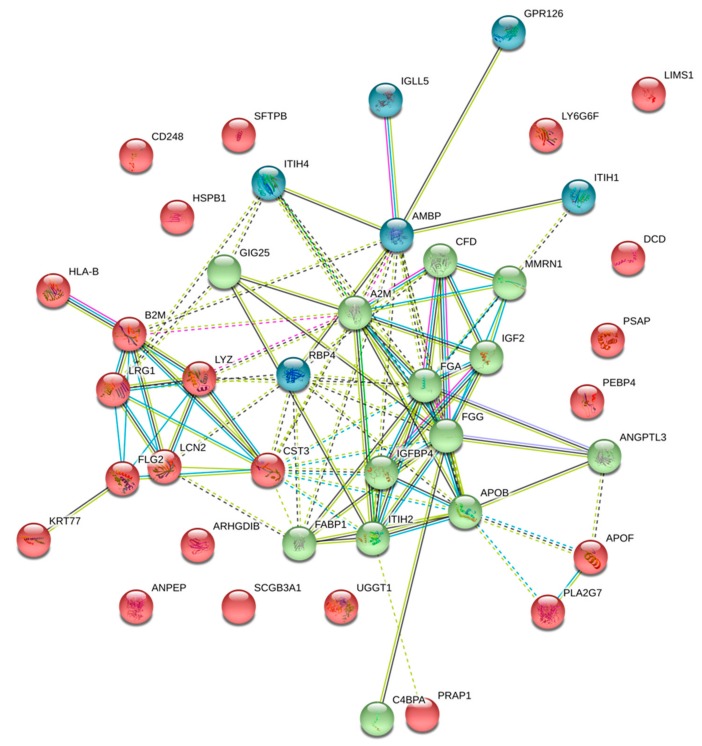
STRING protein–protein interaction network of upregulated proteins in HDL from HD patients. Proteins with a HD/CTL ratio ≥1.5 were analyzed with the STRING bioinformatic tool (https://string-db.org). Three clusters were identified using a k-means approach. Those clusters are highlighted in three different colors. Nodes represent proteins and lines of interactions between proteins.

**Figure 3:**
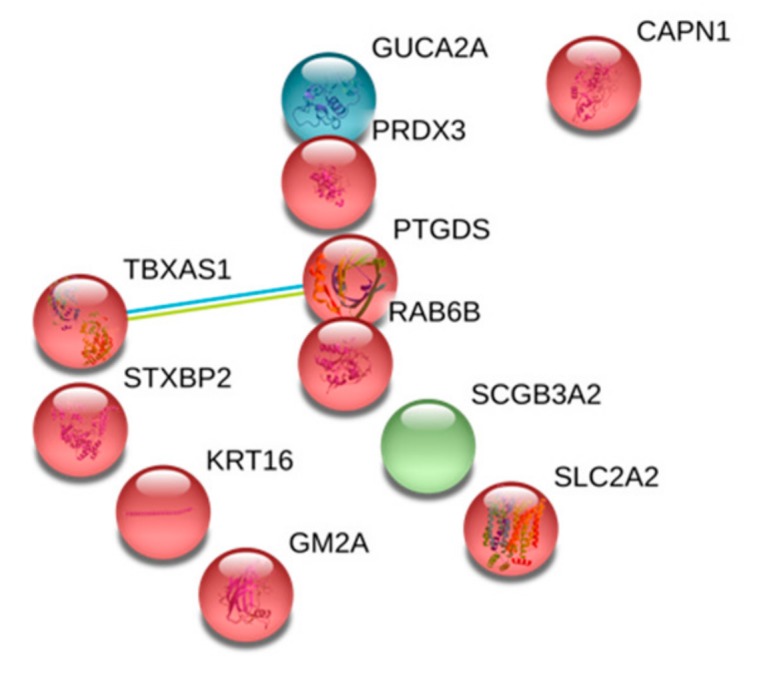
STRING protein–protein interaction network of downregulated proteins in HDL from HD patients. Proteins with a HD/CTL ratio ≤0.66 were analyzed with the STRING bioinformatic tool (https://string-db.org). Three clusters were identified using a k-means approach. Those clusters are highlighted in three different colors. Nodes represent proteins and lines of interactions between proteins.

Gene ontology enrichment analysis of the 46 proteins with a HD/Control ratio ≥1.5 is shown in Figure 2. These proteins were mainly involved in the regulation of lipid metabolism and transport, activation of the immune response, coagulation and platelet activation processes, upregulation of the renewal of the extracellular matrix, generation of reactive oxygen species, and inhibition of apoptosis (Table S4). Gene ontology enrichment analysis of the 11 proteins with a HD/Control ≤0.66 is shown in Figure 3. Those proteins were involved in a cyclooxygenase pathway, a prostaglandin metabolic process, and a fatty acid derivate biosynthetic process (GO:0019371, GO:0006693, and GO:1901570, respectively).

Proteins only found in control samples are listed in Table S2; gene ontology enrichment analysis showed that they mainly belonged to immune response pathways. Proteins only found in HD samples are listed in Table S3; gene ontology enrichment analysis did not find relevant pathways from these proteins.

### 2.2. HD-Associated Protein Expression Correlations in HD Patients

**Figure 4:**
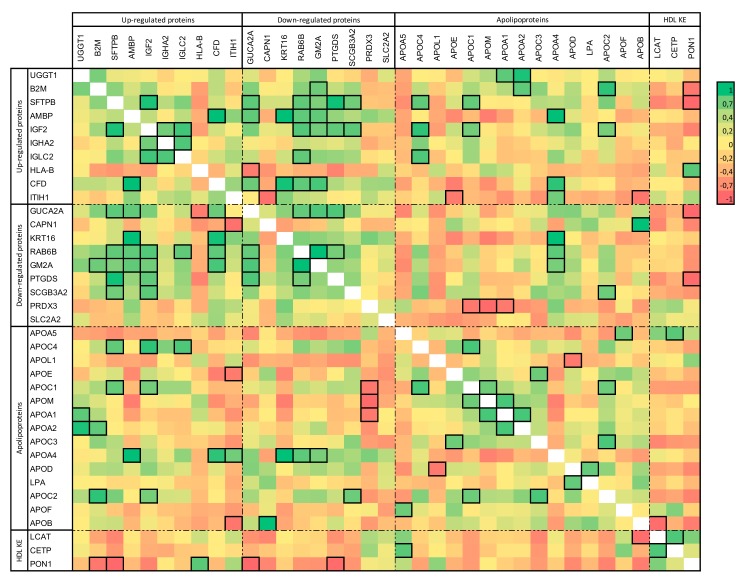
Correlation matrix of up- and downregulated proteins from HDL from HD patients, and apolipoproteins and key-enzymes of HDL. A positive correlation is represented in green and a negative correlation in red. A significant correlation between two proteins is framed. *P* < 0.05 was considered as significant (multiple Spearman correlation matrix).

Ratios of up- and downregulated proteins, apolipoproteins, and key-enzymes in HDL metabolisms were expressed as each HD patient ratio versus mean control ratios. The results from the correlation analysis are represented as a correlation matrix in Figure 4.

Among these correlations, Apolipoprotein E and B (APOE and APOB) amounts were significantly negatively correlated with the inter-alpha-trypsin inhibitor heavy chain H1 (ITHI1) amount in HDL. The paraoxonase 1 (PON1) amount was also negatively correlated with beta-2-microglobulin (B2M), pulmonary surfactant-associated protein B (SFTPB), and prostaglandin-H2 D-isomerase (PTGDS) abundance in HDL.

## 3. Discussion

We found that the HDL proteome from HD patients contained certainly more than 300 proteins (Figure S1), among which 19 were up- or downregulated when compared to control subjects. Using a high-resolution mass spectrometer, we were able to identify 10 times more proteins associated with HDL than any previous work in the field had identified [8,14]. This finding is consistent with a study conducted in chronic heart failure patients, where the authors reported 494 proteins associated with HDL in this pathological context [15]. Simultaneously, our findings were consistent with the HDL proteome watch database (see Table S1, http://homepages.uc.edu/~davidswm/HDLproteome.html). This highlights not only the complexity of the HDL structure but possibly other lipoproteins with diverse roles in pathophysiological processes, such as those observed in CKD. In hemodialysis patients, many beneficial properties of HDL, such as cholesterol efflux, anti-oxidant, and vasodilatory effects, have been found to be impaired [5,6,7,8]. Gene ontology enrichment analyses of our dataset indicated that dysregulated HDL protein composition in HD patients likely underlies the altered properties of HDL observed in this population. Upregulated proteins were involved in extracellular matrix expansion and blood coagulation, platelet activation, oxidative stress, activation of immune system, and negative regulation of apoptosis. Taken together, the dysregulation of these proteins could point to poor vascular homeostasis, which would then contribute to endothelial dysfunction and premature aging of blood vessels.

In a previous study also using label-free quantification, Mangé et al. found 19 upregulated and 21 downregulated proteins in HDL from HD patients [14]. Among these, only two proteins, ß2-microglobulin and AMBP protein, were also found to be similarly dysregulated in our work. Moreover, we did not confirm the finding by Holzer et al. that ApoA1 and Apo A2 are negatively correlated with serum albumin, serum amyloid A, and ApoC3 in HDL particles from HD patients [8]. According to Shao et al., AMBP, B2M, CFD, CST3, RBP4, and PTGDS were enriched in HDL from ESRD patients [13]. Although AMBP and B2M were also found to be enriched in our study, PTGDS was shown to be downregulated herein. This latter protein is associated with renal damage, and its concentrations rise while the glomerular filtration rate (GFR) declines, especially in diabetes mellitus patients. In the present study, we only explored the protein cargo of HDL in HD patients that cannot actually be considered as an index of plasma concentration of this protein. Two factors could, however, account for the downregulation of PTGDS observed in our cohort. First, the patients included in the present study were all non-diabetic in contrast to the study by Shao et al. (in which 55% of patients had diabetes). Second, among our nine HD patients, six were anuric. This status is unfortunately not documented in the Shao et al. study [13] and could be correlated with PTGDS levels in HDL. PTGDS downregulation could be related to the residual renal function (RRF), as this protein has been reported to be an interesting marker for estimated GFR calculation in HD patients [16]. In our cohort, we noticed that all patients with an RRF exhibited a PTGDS ratio >1, while all anuric patients exhibited a PTGDS ratio <1 (Fisher’s exact test, *p* = 0.012) (see Supplementary Table S5). A major difference between the study herein and previous ones is the choice of HD patients. Most patients in the aforementioned studies were diabetic, a situation that could per se lead to significant differences in HDL proteome composition. Patients included in two of those studies were also older than patients included herein. Most importantly, quantification methods, false discovery rate (FDR) threshold, and baseline for significant change in protein expression were different in all studies.

The potential critical role of AMBP protein in lipid and metabolic dysregulation in CKD is confirmed here. The upregulation of AMBP reported in our analysis is in line with the findings from both Mangé et al. and Shao et al. Interestingly, a proteomic analysis of adipose tissue from HD patients also revealed an upregulation of AMBP [17]. AMBP is the precursor of alpha-1-microglobulin, a protein involved in the scavenging and metabolism of free radical and oxidizing residues. Its upregulation could thus be linked to the increase in oxidative stress observed in HD patients [17]. Our results show correlations between the expressions of some HDL key metabolic proteins and the observed dysregulated proteins (Figure 4). Among those correlations, levels of ITHI1, PRDX3, SFTPB, and B2M were strongly associated with key known proteins of HDL expression. Those proteins are involved in different pathways and their clinical and biological relevance in HDL composition remains unknown. However, the correlation between B2M and PON1 levels strengthens the deep implication of B2M in cardiovascular complications in hemodialysis patients. The proteomic approach can indeed help to determine highly sensitive and resolutive patterns of protein expression, but these should be interpreted with caution.

### Limitations of the Study

Beyond the effects of CKD on proteome composition, the altered protein expressions observed herein can also be explained by the hemodialysis procedure per se. Indeed, the hemodialysis procedure is responsible for bio-incompatibility mechanisms including activation of coagulation and immune system processes (complement activation, neutrophil degranulation) that could lead to a specific enrichment of the HDL proteome. This potential contamination process could in turn also be responsible for the disturbances in HDL normal properties. Otherwise, HDL proteome composition could be parasitized by plasma proteins that stricto sensu do not belong to HDL particles. However, the two sequential ultracentrifugations with an adjusted density using potassium bromide seriously limit such contamination. Some other factors such as age or therapies could have modified the protein cargo of HDL in our patients. Nevertheless, the majority of our HD patients were under statins, mitigating the potential effects of such medicine on the proteome in our cohort. Finally, as the number of included subjects was rather low and as we do not include a validation cohort, our study remains exploratory and extrapolations should be made with caution.

## 4. Conclusions

Alteration of the HDL proteome composition of HD patients could underlie HDL dysfunction among this population, leading to the increased cardiovascular risk observed in those patients [1]. Future studies need to focus on the relevance and impact of such protein dysregulation on HDL properties to provide insights on how to restore normal HDL function in CKD and HD patients.

## 5. Materials and Methods

### 5.1. Recruitment and Sample Collection

Patients were sampled at the Lyon teaching hospitals (Hospices civils de Lyon, Hôpital E. Herriot, Lyon, France). Control patients were healthy volunteers referred to the renal unit for a living kidney donation and who were hospitalized in the context of a pre-donation laboratory and clinical work-up. HD patients were recruited within the hemodialysis unit before the mid-week session. Patients aged ≥18 years and undergoing hemodialysis for more than six months were included. Subjects with diabetes mellitus, ongoing inflammatory disease, liver cirrhosis, a recent (less than three months’ ago) cardiovascular event (i.e., myocardial infarction, stroke, acute peripheral artery occlusion), uncontrolled anemia, coagulopathy, and BMI ≥35 kg·m^−2^ were excluded. The study was conducted in accordance with the Declaration of Helsinki and was approved by the institutional review board (CPP Lyon Sud Est IV, ref: L16-57). Written informed consent was obtained from all subjects. Blood samples were obtained by venipuncture on EDTA-coated tubes. Blood samples were centrifuged at 3500 g for 10 min to isolate plasma which was then stored at −80 °C until use. Lipoproteins were separated from plasma by stepwise potassium bromide (KBr) ultracentrifugation as described by Havel et al. [18]

### 5.2. Mass Spectrometry

Briefly, samples were reduced, alkylated, and digested with trypsin at 37 °C overnight. They were desalted on C18 spin columns, dried, and analyzed in triplicate using an Ultimate 3000 nano-RSLC (Thermo Fisher Scientific, Illkirch, France) coupled online to a Q-Exactive HF hybrid Quadrupole-Orbitrap Mass Spectrometer (Q-Orbitrap, Thermo Fisher Scientific). Briefly, peptides were separated on a C18 nano-column using an acetonitrile linear gradient and analyzed in a Top 20 Higher Collision Dissociation (HCD) data-dependent mass spectrometer. Data were processed by database searching using Sequest HT (Thermo Fisher Scientific) with Proteome Discoverer 2.2 software (Thermo Fisher Scientific) against a human Swissprot database and quantified with a label-free quantification approach. The quantification was expressed either as a ratio of the mean relative amount of protein in HD samples versus the mean relative amount in control samples, or the relative amount in each HD sample versus the mean relative amount in control samples. Precursor and fragment mass tolerances were set at 10 ppm and 0.02 Da, respectively. Trypsin was set as the enzyme, and up to two missed cleavages were allowed. Peptides were filtered with FDR at 1% with a Benjamini–Hochberg correction.

### 5.3. Gene Ontology and Protein Network

Gene ontology enrichment and protein network analysis of the data was done using the STRING tool (https://string-db.org). For these analyses, all proteins with a ratio of ≥1.5 and ≤0.66 were included. Enrichments with an FDR lower than 5% were considered significant.

### 5.4. Statistical Analysis

Data are expressed as mean ± standard error of the mean (SEM). All analyses were performed using GraphPad Prism version 6.0 (GraphPad software, La Jolla, CA, USA) and XLSTAT software (Addinsoft. XLSTAT 2016: Data Analysis and Statistical Solution for Microsoft Excel. Paris, France). Multiple correlations were made using Spearman’s rank correlation, and the correlation matrix was presented as a heatmap. Protein quantitation was performed with a Minora feature detector and precursor ions quantifier node in Proteome Discoverer 2.2 software with protein quantitation based on pairwise ratios and the ANOVA (individual proteins) hypothesis test. Differences were considered as significant at the *p* < 0.05 level.

### 5.5. Associated Data

The mass spectrometry proteomics data have been deposited to the ProteomeX change Consortium via the PRIDE partner repository with the dataset identifier PXD013301 (DOI: 10.6019/PXD013301, username: reviewer26456@ebi.ac.uk, password: oMD4gyDT).

## Figures and Tables

**Table 1:** Characteristics of hemodialysis and control patients.

N	Control (CTL)	Hemodialysis (HD)	*P*-value
	8	9	
**General characteristics**			
Age, years	39 (31–50)	57 (46–74)	0.045
Gender, *n* male/*n* female	5/3	5/4	0.653
BMI, kg/m^2^	22 (19–26)	26 (25–28)	0.060
*Comorbidities*			
HT, *n*	2	8	
Stroke, *n*	0	0	
CHD, *n*	0	2	
Cardiopathy, *n*	0	4	
PVD, *n*	0	1	
*Therapies*			
Statins, *n*	0	6	
PI, *n*	0	5	
RASi, *n*	1	3	
ß-blockers, *n*	1	5	
CCB, *n*	1	1	
**Biological parameters**			
Urea, mmol/L	6.5 (5.3–7.8)	13.0 (10.9–19.8)	<0.0001
Creatinine, µmol/L	77.5 (5.3–7.8)	583 (458.0–798.0)	<0.0001
mGFR, mL/min/1.73 m^2^	94 (84–96)	-	
Total cholesterol, mg/dL	217 (187–238)	153 (104–191)	0.021
LDL cholesterol, mg/dL	141 (104–157)	71 (43–124)	0.029
HDL cholesterol, mg/dL	58 (52–62)	46 (38–48)	0.016
Triacylglycerols, mg/dL	99 (86–133)	93 (87–138)	0.999
CRP, mg/L	1.7 (0.2–4.6)	2.5 (1.5–24.8)	0.145

Data are expressed as median (interquartile range). BMI: body mass index, HT: hypertension, CHD: coronary heart disease, GFR, glomerular filtration rate, PVD: peripheral vascular disease, PI: platelet inhibitor, RASi: renin-angiotensin system inhibitor, CCB: calcium-channel blocker, mGFR: measured glomerular filtration rate by iohexol clearance, CRP: C-reactive protein. Creatinine: × 0.011 for mg/dL, urea: × 2.8 for mg/dL. Differences were considered significant at the *P* < 0.05 level (Mann–Whitney U tests).

**Table 2:** List of upregulated proteins in high-density lipoprotein (HDL) from hemodialysis patients.

Protein Name	Protein Label	Ratio	*P*-value
UDP-glucose: glycoprotein glucosyltransferase 1	UGGT1	3.948	9.34 × 10^−6^
Beta-2-microglobulin	B2M	2.895	7.90 × 10^−4^
Pulmonary surfactant-associated protein B	SFTPB	2.716	1.72 × 10^−3^
Protein AMBP	AMBP	2.711	1.75 × 10^−3^
Insulin-like growth factor II	IGF2	2.672	2.07 × 10^−3^
Immunoglobulin heavy constant alpha 2	IGHA2	2.615	2.66 × 10^−3^
Immunoglobulin lambda constant 2	IGLC2	2.514	4.13 × 10^−3^
HLA class I histocompatibility antigen, B-58 alpha chain	HLA-B	2.427	6.05 × 10^−3^
Complement factor D	CFD	2.224	1.46 × 10^−2^
Inter-alpha-trypsin inhibitor heavy chain H1	ITIH1	2.073	2.79 × 10^−2^

Protein ratio was calculated as protein abundance in HD patients/protein abundance in control.

**Table 3:** List of downregulated proteins in HDL from hemodialysis patients.

Protein Name	Protein Label	Ratio	P-value
Guanylin	GUCA2A	0.553	1.71 × 10^−2^
Calpain-1 catalytic subunit	CAPN1	0.538	1.32 × 10^−2^
Keratin, type I cytoskeletal 16	KRT16	0.526	1.05 × 10^−2^
Ras-related protein Rab-6B	RAB6B	0.519	9.23 × 10^−3^
Ganglioside GM2 activator	GM2A	0.513	8.19 × 10^−3^
Prostaglandin-H2 D-isomerase	PTGDS	0.458	2.40 × 10^−3^
Secretoglobin family 3A member 2	SCGB3A2	0.424	9.51 × 10^−4^
Thioredoxin-dependent peroxide reductase, mitochondrial	PRDX3	0.404	5.22 × 10^−4^
Solute carrier family 2, facilitated glucose transporter member 2	SLC2A2	0.251	3.16 × 10^−7^

Protein ratio was calculated as protein abundance in HD patients/protein abundance in control.

## Supplementary Materials

The following are available online at https://www.mdpi.com/2072-6651/11/11/671/s1, Figure S1: STRING protein–protein interaction network of all proteins in HDL from HD patients, Table S1: protein list and mass spectrometry analysis statistics, Table S2: Proteins only found in Control samples, Table S3: Proteins only found in HD samples, Table S4: Gene ontology enrichment of upregulated proteins (HD/CTL ratio greater than 1.5), Table S5: Reduced PTGDS abundance in anuric HD patients.
