# Supplementary material for: Proteomic Characterization of High-Density Lipoprotein Particles from Non-Diabetic Hemodialysis Patients
Source: Toxins (Basel). 2019 Nov 15;11(11):671. doi: 10.3390/toxins11110671 (PMC6891510; doi:10.3390/toxins11110671)
Supplement: Supplementary file 1 [file toxins-11-00671-s001.zip › Supplementary_files_R1/Table S5.pdf]

**Supplementary Table S5**<br>**Reduced PTDGS abundance in anuric HD patients**		
	**PTGDS ratio (HD/CTL)**	
	**Lower than 1**	**Higher than 1**
**Anuric**	6	0
**Diuresis >100 cc**	0	3
Fisher's exact test, p=0.012		
