# Supplementary figures and images for: Proteomic Characterization of High-Density Lipoprotein Particles from Non-Diabetic Hemodialysis Patients
Source: Toxins (Basel). 2019 Nov 15;11(11):671. doi: 10.3390/toxins11110671 (PMC6891510; doi:10.3390/toxins11110671)

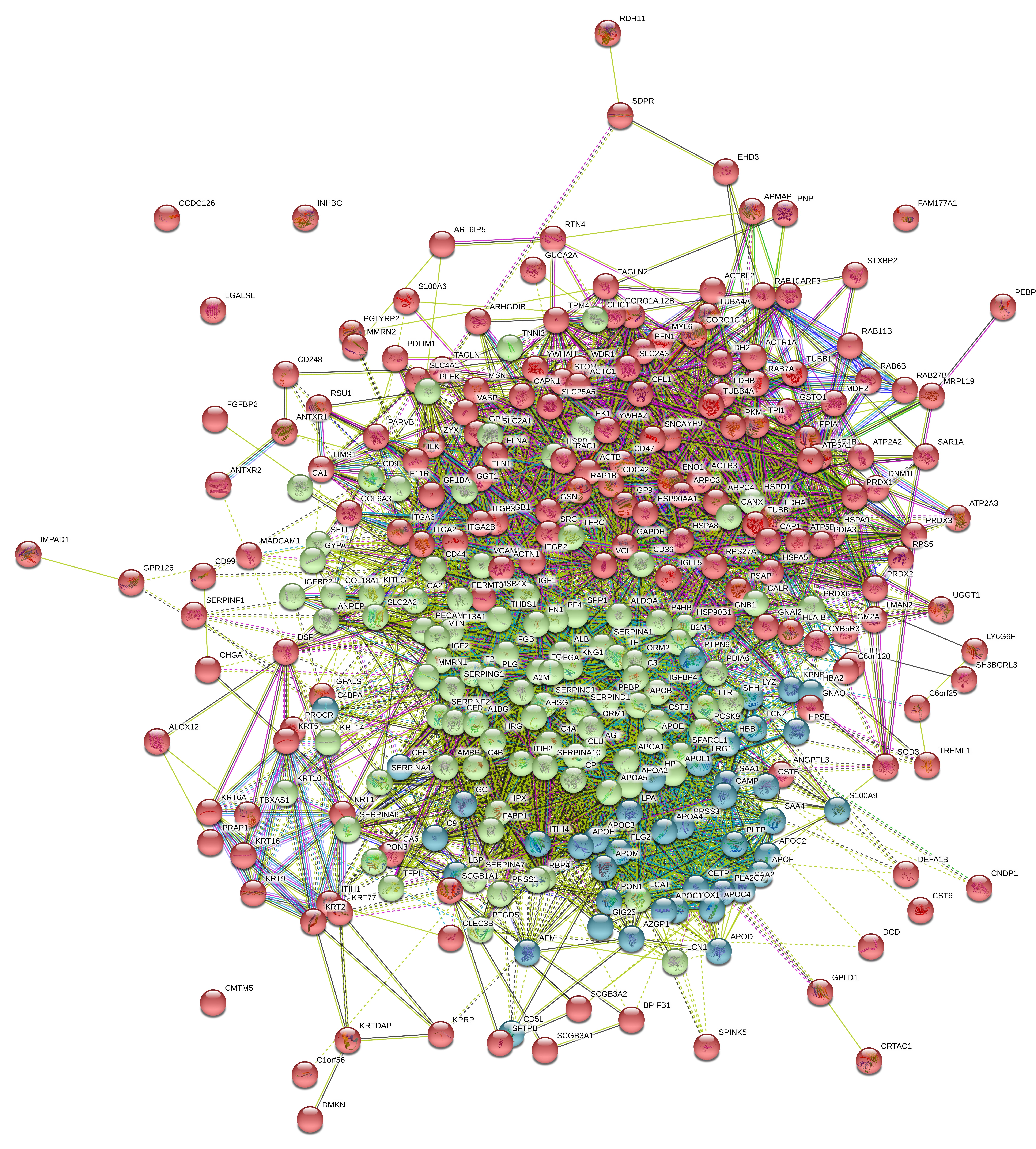

Supplement: Supplementary file 1 [file toxins-11-00671-s001.zip › Supplementary_files_R1/figure S1.jpg]
